# The Psychometric Properties of the Type 1 Diabetes Mellitus Screening Acceptability Assessment (DMSA) Scale among General Population

**DOI:** 10.1155/2024/1286029

**Published:** 2024-07-25

**Authors:** Iman S. Al-Gadi, Amirah D. Albalawi, Reem A. Al Khalifah

**Affiliations:** ^1^Division of Pediatric Endocrinology, Department of Pediatrics, College of Medicine, King Saud University, Riyadh 11461, Saudi Arabia; ^2^The University Diabetes Centre, King Saud University Medical City, King Saud University, Riyadh 11461, Saudi Arabia

## Abstract

**Background:**

Type 1 diabetes mellitus (T1DM) screening facilitates access to early intervention and prevention of severe complications, such as diabetic ketoacidosis. Despite its significance, many countries lack a systematic T1DM screening program. Understanding how the public perceives T1DM screening for children is essential for successfully implementing such programs but is currently an area with limited research. Our study aims to fill this gap by developing a standardized tool designed to assess the acceptability of T1DM screening programs for children, focusing on caregiver perspectives within the general population.

**Materials and Methods:**

We developed the Type 1 Diabetes Mellitus Screening Acceptability (DMSA) scale based on the theoretical framework of acceptability and integrated components from the Pediatric Testing Attitudes Scale-Diabetes (P-TAS-D). It covers a broad spectrum of acceptability constructs. The DMSA scale underwent iterative modifications following expert feedback to refine clarity and content validity. We tested the scale in both Arabic and English with adults living in Saudi Arabia, regardless of their parental status, focusing on the potential of screening their children. The psychometric strengths of the scale were evaluated through reliability analyses and exploratory factor analysis.

**Results:**

Of the 599 participants, the majority were female (89.2%), with a mean age of 35.9 ± 8.6 years. The final DMSA scale consists of 10 items, with two distinct factors: “individual acceptability” and “psychosocial acceptability.” The mean total score was 42.9 ± 5.1 across a potential range of 10–50 points. The English and Arabic versions of the scale demonstrated strong reliability, with Cronbach's alpha values of 0.84 and 0.79, respectively.

**Conclusions:**

The DMSA scale emerges as a valid and reliable tool for gauging the acceptability of the general population of screening children for T1DM. It integrates key elements of the acceptability construct, pivotal for guiding the implementation of culturally sensitive T1DM screening initiatives. Future research should expand its application across various cultural settings and examine the correlation between scale scores and actual screening behaviors.

## 1. Introduction

Type 1 diabetes mellitus (T1DM) imposes a significant global health burden, affecting individuals, families, and healthcare systems worldwide. In Saudi Arabia, the incidence of T1DM among youth has risen to 31.4 cases per 100,000 individuals, a nearly ninefold increase over the last decade [1, 2]. Pancreatic islet antibody testing presents a promising method for predicting T1DM risk in asymptomatic children, with positive results indicating a greater than 80% likelihood of progression to stage 3 T1DM within 15 years [3]. Early detection through islet antibody screening allows for early intervention and mitigation of complications [4, 5]. Screening significantly reduced the incidence of diabetic ketoacidosis (DKA) at disease onset across studies and settings. Additional benefits include earlier diagnosis, timely education, and potential long-term benefits, such as lower HbA1c levels [5, 6, 7] Despite these advantages, T1DM screening also has potential drawbacks, including increased anxiety and stress among parents and children, potential impacts on quality of life, and the need for regular follow-ups and possible overmedicalization of at-risk individuals [5, 6].

T1DM screening programs are established in North America and Europe but are notably absent in Saudi Arabia and other Arab countries [5, 6]. There is an ongoing global debate regarding the screening scope, timing, and type of approach [6, 8, 9]. The acceptance of such programs by the general population is a critical consideration for their implementation. The evaluation of acceptability is crucial at various stages, including the design, assessment, and execution phases of any healthcare intervention. However, it remains understudied, with no available targeted and standardized tool to assess the general population's acceptability of programs screening children for T1DM [10, 11, 12, 13]. Assessing the acceptability of an intervention is complex as it reflects individuals' perceptions of its appropriateness based on expected or experienced cognitive and emotional responses [14]. The theoretical framework of acceptability consists of seven constructs that offer a structured approach for determining the acceptability of a healthcare intervention, such as T1DM screening programs [15].

Our study aims to bridge this gap by developing a Type 1 Diabetes Mellitus Screening Acceptability (DMSA) scale, leveraging the theoretical framework of acceptability [15]. Our goal in developing and testing the psychometric properties of this tool is to enable a comprehensive assessment of population attitudes toward T1DM screening for children from a caregiver perspectives. These insights will empower stakeholders with valuable evidence-based information to help guide the future development of culturally sensitive and sustainable screening strategies tailored to the unique needs and preferences of the population.

## 2. Materials and Methods

### 2.1. Design and Participants

We conducted a cross-sectional study from January to February 2023, using a nationwide self-administered survey targeting adults over 18 years old residing in Saudi Arabia. The focus was on the potential of screening their children, regardless of their parental status. The survey was self-administered online using the Redcap platform. It was distributed through multiple channels, such as the pediatric diabetes and endocrinology clinics across the country, the physician mailing list of the Saudi Pediatric Association, and different social media platforms, including Twitter, WhatsApp, Snapchat, Instagram, and LinkedIn. The study received ethical approval from the King Saud University Institutional Review Board, and all participants gave online informed consent.

### 2.2. DMSA Scale Development

#### 2.2.1. Conceptualization and Item Generation

We used the theoretical framework of acceptability to create a method for evaluating the acceptability of T1DM screening programs in the general population, incorporating elements from the Pediatric Testing Attitudes Scale-Diabetes (P-TAS-D). The theoretical framework of acceptability includes seven constructs: affective attitude, burden, ethicality, intervention coherence, opportunity costs, perceived effectiveness, and self-efficacy [14]. A generic tool based on this framework was developed to evaluate the acceptability of healthcare interventions and serves as a valuable source for recognizing aspects of interventions that might be enhanced [15]. However, adapting it for screening interventions poses challenges, particularly as T1DM is predominantly understood by those directly affected rather than the general population, hindering comprehension of potential consequences and therapeutic options of T1DM by the general population [10, 15]. The P-TAS-D is an 11-item scale designed to evaluate parental attitudes toward T1DM risk screening in children, involving the three domains: attitudes and beliefs, communication about risk screening results, and decision making, but lacks other vital constructs of intervention acceptability [13].

Thus, we developed the DMSA scale drawing from the existing literature on the theoretical framework of acceptability and P-TAS-D. The DMSA constructs encompass affective attitude, ethicality, perceived effectiveness, self-efficacy, opportunity costs, decision-making, communication regarding the program, intervention coherence, general acceptability, communication of results, psychosocial burden, and cultural acceptability. The original version of the DMSA scale had 12 items, each structured based on the tested domain and tailored for T1DM screening considering the clinical uncertainty of screening and lack of T1DM cure. We also incorporated additional questions outside of the scale to explore reasons for screening rejection intended to offer insights for policymakers. Scale items were designed to test caregiver acceptability of screening their children for T1DM. Items were positively worded and evaluated for readability at a Flesch–Kincaid grade 6 English level. Each item was scored on a Likert scale ranging from 1 to 5, where a score of 5 indicated complete agreement and a score of 1 indicated complete disagreement. However, items Q10 and Q11 (numbered based on the final version) were reverse-scored.

#### 2.2.2. Face and Content Validation

The DMSA scale items underwent rigorous face and content validation by a panel of 20 individuals, including 17 diabetes experts consisting of eight pediatric endocrinologists, one adult endocrinologist, one psychiatrist, one psychologist, two nurses, and four diabetic educators. Furthermore, input was provided by three parents. The panel evaluated the scale through an online survey. They were asked to assess the Arabic version of each item for its content validity and clarity, rating them on a scale from 0 to 100, and offering feedback on any ambiguous items or proposing modifications. All items had a median score of more than 80 for appropriateness and clarity, except for one. As a result, the panel removed this item, which targeted the intervention coherence construct: “The expected benefit of early screening for T1DM in children outweighs the potential harm” due to concerns about its content validity and clarity. Multiple iterative revisions were made to the scale items based on expert feedback to enhance clarity and content validity. The tested version included 11 items (Table 1). The Arabic version was adapted for validation by translating it to English and back-translating it to Arabic to ensure the items retained their original meanings and were appropriate for the target population. The scale was designed for online self-administration in either Arabic or English to minimize potential social desirability bias. Before consenting to study participation, the participant was required to read a script explaining T1DM, its treatment, screening opportunities, and potential therapies to delay disease onset (Supplementary Materials (available here)).

### 2.3. Additional Measures

We collected demographic data on age, sex, income for socioeconomic status, and personal history of diabetes. We also used the Generalized Anxiety Disorder-7-item scale (GAD-7) to evaluate baseline anxiety in caregivers and its association with screening refusal. The GAD-7 is a simple tool used for the initial screening of generalized anxiety disorder. It consists of seven items that evaluate anxiety symptoms over the past 2 weeks using a 4-point scale. A score of 8 or higher suggests possible generalized anxiety disorder, with a sensitivity of 92% and specificity of 76% [16].

### 2.4. Outcomes

We assessed the reliability of the DMSA scale using Cronbach's alpha coefficient and item-total correlation for internal consistency. Additionally, we conducted face and content validation of the DMSA scale as described above. Construct validity was evaluated through factor analysis, while discriminant validity was determined by correlating the DMSA scale with the GAD-7 scale.

### 2.5. Statistical Analysis

We summarized baseline characteristics as mean with SD for continuous data, median with interquartile range for skewed data, and *N* (%) for dichotomous data. We presented statistics of individual DMSA scale item scores using mean and SD. We assessed internal consistency using Cronbach's alpha for items and the total DMSA scale separately for the English and Arabic versions. A Cronbach's alpha value of 0.70 or higher was considered acceptable [17, 18]. We also conducted item analysis for internal consistency reliability using item-total correlations to evaluate the correlation between each item and the total DMSA scale. This was performed separately for the English and Arabic versions of the DMSA scale. We dropped items from the scales if the item-total correlations were at a cut-off of ≤0.2, defining discriminating items [17].

We assessed the validity of the DMSA scale through various measures. Construct validity was examined using exploratory factor analysis with oblique promax rotation to identify scale dimensions. We chose the number of factors based on various statistical criteria, including an elbow appearance on a scree plot, factor eigenvalues greater than 1, and a significant likelihood ratio test. We examined factor loading and unique variances for each item. We determined discriminant validity through univariate linear regression, with DMSA scale scores as the dependent variable and individual income, sex, age, educational level, history of diabetes, and anxiety (GAD-7 scores) as independent variables. A significant association was defined as *p*-value < 0.05. We performed all data analysis using Stata/SE 16.0 for Mac.

## 3. Results

### 3.1. Demographics and Sample Characteristics

A total of 926 participants accessed the online survey, with 607 initiated the survey, of which 599 completed it (553 Arabic and 46 English respondents). The majority were female, 538 (89.2%), with a mean age of 35.9 ± 8.6 years (Table 2). About 8.6% (51) reported having a child with T1DM, and 6.3% (38) reported having T1DM. Participants represented varying income levels. The mean GAD-7 total score was 6.6 ± 5.3 points.

### 3.2. Item Characteristics and Reliability

The mean score for each item ranged between 2.9 and 4.8. The SD of scores displayed variable ranges of responses, representing a desired variability in acceptability within our sample (Table 3). All scale items had an acceptable item-total correlation above 0.2, except for item Q6 (English version). Cronbach's alpha of the DMSA scale was 0.82 for the English version and 0.75 for the Arabic version.

### 3.3. Construct Validly: Factor Analysis

**Figure 1:**
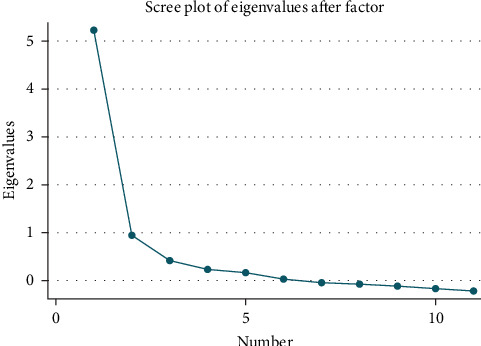
Scree plot analysis for factor structure of the DMSA scale.

Exploratory factor analysis was conducted on the DMSA scale, revealing two principal components with eigenvalues ≥ 1 that explained 99% of the total variance (Figure 1). This two-factor model was further supported by an inflection point observed on the scree plot and a significant likelihood ratio test (chi-square (55) = 3176.98; *p* < 0.0001). The two factors have good face validity, and based on their component items, they were given provisional titles (Table 4). Factor 1, “individual acceptability,” included nine items and accounted for a substantial portion of the variance at 86.6%. Factor 2, “psychosocial acceptability,” included two items that explained 16.7% of the variance.

However, it was noted that item Q6, which pertains to family autonomy in decision-making regarding T1DM risk screening, exhibited a high degree of uniqueness (0.81–0.91) with negative loading. These findings, along with the suboptimal item-total correlation, indicate that Q6 may not be consistent with the overall scale nor relevant within the factor model. Therefore, we excluded the Q6 item from the final scale.

### 3.4. Final DMSA Scale Characteristics

The final DMSA scale has 10 items, with a mean total score of 42.9 ± 5.1 points and a range of a minimum of 10 to a maximum of 50 points. The adjusted scale reflects a more reliable measure of T1DM screening acceptability, as indicated by the higher Cronbach's alpha (0.84 for the English version and 0.79 for the Arabic version). The scale has two subscales, one measuring individual acceptability with a Cronbach's alpha of 0.9 and the other measuring psychosocial acceptability with a Cronbach's alpha of 0.7.

### 3.5. Discriminant Validity

Univariate regression analysis was conducted to investigate the impact of participant characteristics on the total scores of the DMSA scale (Table 5). Results indicated that participants' anxiety levels, as measured by the GAD-7 score, and personal history of diabetes did not significantly affect the acceptability scores ((*B* = 0.006, 95% CI: [−0.07, 0.10], *p*=0.88) and (*B* = 0.5, 95% CI: −0.17, 1.25, *p*=0.14), respectively). This observation holds true for personal history of T1DM compared to other types of diabetes (*B* = 0.83, 95% CI: [−1.61, 3.2], *p*=0.5). However, a notable reduction in the acceptability scores was observed with increasing income, declining by 0.4 points for every 5,000 SR increment in monthly income (95% CI: −0.64, −0.25, *p*  < 0.001).

Additionally, a subgroup analysis of the DMSA subscales showed that the individual acceptability subscale scores were positively influenced by the psychosocial acceptability subscale scores and personal history of diabetes. The individual acceptability subscale scores increased by 0.33 points with every 1-point increment in the psychosocial acceptability subscale (95% CI: 0.15, 0.50, *p* < 0.001), and 0.67 points for individuals with any type of diabetes (95% CI: 0.45, 1.29, *p*  < 0.04). When examining if the history of T1DM differed in effect on the DMSA subscales compared to other types of diabetes, there was no significant difference in the individual acceptability subscale scores between participants with a personal history of T1DM (37.18 ± 4.23) and those with Type 2 diabetes (36.48 ± 5.48).

### 3.6. Perception on Mental Health and Social Views

Around 40% of participants expressed that the prospect of screening their child for T1DM might adversely affect their mental health (Q10), and 24% endorsed apprehension that a positive early screening result for their child might lead to negative societal perceptions toward their family (Q11). However, despite these apprehensions, the average total DMSA score for those participants was 40 ± 5 points, indicating an acceptability score of 80%. The cited reasons behind concerns surrounding T1DM risk screening included the following reasons: increased mental stress for family members, potential restrictions on the child, and change in how the child or family are treated at school or work.

## 4. Discussion and Conclusions

Our study represents a pioneering step in assessing the acceptability of the general population in performing T1DM risk screening by developing the DMSA scale tool, available in both Arabic and English. This bidimensional scale, as indicated by factor analysis, clearly delineates between individual and psychosocial acceptability and covers a wide range of constructs rooted in a robust framework. Its simplicity, ease of administration, and strong display of internal consistency and validity reflect its relevance for the target population.

The observed significant positive association between the individual acceptability of the DMSA scale and the participant's diabetes status suggests that personal experience with the disease may predispose individuals to value early detection more, reflecting heightened awareness of diabetes burden. This aligns with prior research indicating that familiarity with a condition can enhance appreciation and acceptability for preventive measures [10].

Our findings shed light on the psychosocial dimensions of diabetes screening. Individual acceptability scores were influenced by psychosocial acceptability scores, suggesting potential social desirability bias. Moreover, a substantial portion of participants worried about the potential negative impacts on mental health and societal perceptions, which reflects an underlying stigma associated with the diagnosis. Nonetheless, the average acceptability score exceeded 70%, emphasizing the need to assess the association between attained scores and actual screening acceptance or refusal behavior.

An item intended to evaluate the “intervention coherence” construct within the acceptability framework [14] underperformed in terms of content validity, leading to its exclusion from further psychometric analysis. Most of our expert panel adjudged the statement assessing the balance of benefits over harms in early T1DM screening as having minimal relevance to acceptability assessment. Various explanations could underlie this finding, such as the uncertainties among healthcare professionals surrounding the cost-effectiveness and balance between benefits and potential harms of T1DM screening [7, 19], as highlighted in a British study examining stakeholder perspective on pediatric general population T1DM screening [11] and echoed in our findings. Furthermore, the inherent complexity in the intervention coherence construct presents significant challenges in the context of T1DM screening, hindering effective capture through simplistic items.

Furthermore, the “decision-making” construct adapted from the P-TAS-D [13] failed to resonate with the unique cultural or contextual intricacies of our study population. This misalignment may stem from the prevalent paternalistic medical decision-making culture in our setting [20, 21]. Such cultural differences are likely to influence the psychometric attributes of this construct, suggesting it might perform differently in cultures that prioritize autonomy [13].

Our study introduces the DMSA scale as a new tool for assessing T1DM screening acceptability, laying the groundwork for further predictive validation studies in the T1DM screening field. It is intended to facilitate screening uptake and inform policy decisions to overcome barriers to screening on a population level. However, the study has its limitations. While we established excellent internal consistency, additional reliability measures such as inter-rater agreement and test–retest reliability were not assessed. Also, the DMSA scale scores were not validated against actual screening acceptance or refusal behavior, warranting further investigation into its prognostic value in real-world settings where screening is actively offered. Additionally, the cohort consists of 89% highly educated females and only 8% of the questionnaires were in English. Studies are needed to establish reliability measures among male participants and English speakers.

In conclusion, the DMSA scale is a simple, self-administered instrument that has demonstrated reliability and validity when applied to the general population. Its bidimensional nature captures the complex interplay between individual preferences and societal norms. While promising, the scale requires additional validation across varied populations to confirm its universal applicability and effectiveness in different cultural contexts.

## Figures and Tables

**Table 1:** Type 1 Diabetes Mellitus Screening Acceptability (DMSA) scale.

Item	English	Arabic	Tested construct
Q1	I prefer screening my child for Type 1 diabetes to find out how likely it is that they will get it	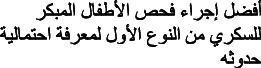	Affective attitude
Q2	I believe it is fair and helpful for the family to have a screening program for Type 1 diabetes risk in children	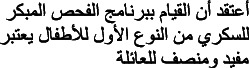	Ethicality
Q3	I believe that early screening of children for Type 1 diabetes risk can improve the chances for receiving early care or intervention	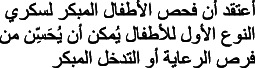	Perceived effectiveness
Q4	I am willing to do whatever is needed for screening my child for Type 1 diabetes (including attending appointments, taking blood samples, and more if needed)	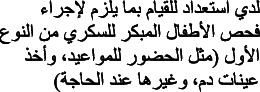	Self-efficacy
Q5	I would like to have my child screened for Type 1 diabetes early, even if I have to pay for it myself	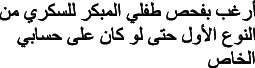	Opportunity costs
Q6 (excluded)	The family has the right to decide whether to check their child's risk for Type 1 diabetes, regardless of the opinion of their treating doctor	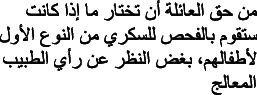	Decision making
Q7	If there is a program for early Type 1 diabetes screening in children, I prefer to find details about it from my child's doctor at an early age	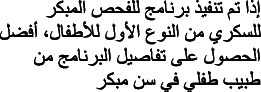	Communication regarding program
Q8	If there is program for early Type 1 diabetes screening program, I will have my children checked	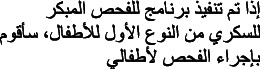	General acceptability
Q9	If an early screening program for Type 1 diabetes in children is carried out, I will share the screening results with my child as appropriate for their understanding	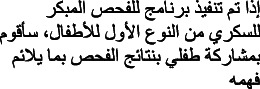	Communication of results
Q10	The process of screening my child for Type 1 diabetes risk may have a negative effect on my mental health	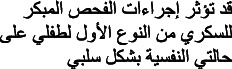	Burden (psychological)
Q11	If early screening for Type 1 diabetes in my child comes back positive, I might feel negatively about how society thinks of my family	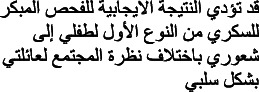	Cultural acceptability

Likert scale scoring for each item: 1 (completely disagree) to 5 (completely agree). All items are positively scored except for Q10 and Q11 are reverse-scored.

**Table 2:** Demographic and clinical characteristics of the study sample.

Variable	Total, *N* (%), 599	Arabic, *N* (%), 553 (92.32)	English, *N* (%), 46 (7.68)	*p*-Value
Age, years	35.89 ± 8.75	35.80 ± 8.44	35.64 ± 10.55	0.91
Sex, female	538 (89.22%)	504 (91.38%)	34 (73.91%)	0.001
Education, level				<0.001
No education	3 (0.50%)	3 (0.54%)	0 (0%)	—
Primary and intermediate	12 (2.01%)	12 (2.19%)	0 (0%)	—
High school and diploma	152 (25.42%)	150 (27.17%)	2 (4.35%)	—
University and above	431 (72.07%)	387 (69.98%)	44 (95.65%)	—
Income, SR				<0.001
<5,000	80 (13.3%)	77 (14.0%)	3 (6.52%)	—
5,000–15,000	319 (53.5%)	309 (56.19%)	10 (21.74%)	—
16,000–25,000	120 (20.1%)	112 (36.91%)	8 (15.22%)	—
26,000–35,000	40 (6.7%)	28 (4.09%)	12 (23.92%)	—
36,000–45,000	15 (2.94%)	13 (2.36%)	2 (4.34%)	—
>46,000	22 (3.65%)	11 (2.0%)	11 (23.91%)	—
Diagnosis of any type of diabetes	77 (12.7%)	71 (12.86%)	6 (13.04%)	<0.001
Type of diabetes				
Type 1 diabetes mellitus	38 (6.2%)	37 (52.11%)	1 (16.67%)	0.12
Type 2 diabetes mellitus	31 (5.1%)	26 (36.62%)	5 (83.33%)	—
Gestational diabetes	2 (0.32%)	2 (2.82%)	0 (0%)	—
Not known	6 (0.9%)	6 (8.45%)	0 (0%)	—
Child with Type 1 diabetes mellitus	51 (8.6%)	51 (9.27%)	0 (0%)	0.03
GAD-7, points	6.69 ± 5.28	6.73 ± 5.27	6.19 ± 5.2	0.51

GAD-7, Generalized Anxiety Disorder 7-item scale. A score of 8 or higher suggests possible generalized anxiety disorder.

**Table 3:** Item-level analysis and Cronbach's alpha coefficients for the DMSA scale.

Item	Mean	SD	Arabic		English	
			Item-total correlation	Alpha	Item-total correlation	Alpha
Q1	4.64	0.71	0.74	0.71	0.85	0.78
Q2	4.68	0.66	0.76	0.71	0.76	0.79
Q3	4.76	0.60	0.68	0.72	0.78	0.79
Q4	4.59	0.75	0.74	0.70	0.86	0.78
Q5	4.22	0.99	0.65	0.71	0.68	0.80
Q6	3.75	1.27	0.34	0.79	0.17	0.86
Q7	4.69	0.55	0.63	0.72	0.75	0.80
Q8	4.60	0.68	0.72	0.71	0.83	0.79
Q9	4.48	0.74	0.65	0.72	0.70	0.80
Q10	2.91	1.22	0.43	0.77	0.36	0.84
Q11	3.40	1.23	0.35	0.79	0.22	0.86
Total scale (including Q6)	46.61	5.37	—	0.75	—	0.82
Total scale (Final scale excluding Q6)	42.89	5.10	—	0.79	—	0.84

**Table 4:** Rotated factor loadings and unique variances for the DMSA scale item.

Item	Arabic			English		
	Factor 1: individual acceptability	Factor 2: psychosocial acceptability	Uniqueness	Factor 1: individual acceptability	Factor 2: psychosocial acceptability	Uniqueness
Q1	0.82	—	0.33	0.82	—	0.23
Q2	0.85	—	0.27	0.78	—	0.38
Q3	0.74	—	0.45	0.84	—	0.30
Q4	0.79	—	0.37	0.92	—	0.16
Q5	0.61	—	0.62	0.69	—	0.47
Q6	—	−0.24	0.91	—	−0.43	0.81
Q7	0.66	—	0.55	0.77	—	0.40
Q8	0.80	—	0.35	0.90	—	0.18
Q9	0.66	—	0.56	0.71	—	0.49
Q10	—	0.64	0.57	—	0.52	0.71
Q11	—	0.64	0.58	—	0.56	0.68

Equamax extraction and oblique promax rotation methods used. Em dash indicates loadings <0.25.

**Table 5:** Univariate regression analysis: impact of participant characteristics on DMSA total scores.

Variable	*B* coefficient	95% CI	*p*-Value	*R* ^2^
Age, years	−0.003	−0.06, 0.04	0.90	0.0004
Sex, female	0.35	−0.97,1.66	0.60	0.0004
Education, *ε*				0.0113
No education	1.92	−3.72, 7.55	0.50	—
High school education	0.05	−2.57, 2.67	0.97	—
University education	−1.15	−3.71, 1.40	0.37	—
Income, per 5,000 SR	−0.4	−0.64, −0.25	<0.001	0.04
Personal history of any diabetes*⁣*^*∗*^	0.5	−0.17, 1.25	0.14	0.01
Personal history of T1DM vs. other diabetes*⁣*^*∗*^	0.83	−1.61, 3.2	0.50	0.006
GAD-7, points	0.006	−0.07, 0.10	0.88	0.003

GAD-7, Generalized Anxiety Disorder 7-item scale; T1DM, Type 1 diabetes mellitus; and *ε*, reference category 12 years of education. *⁣*^*∗*^Any diabetes includes T1DM, Type 2 diabetes mellitus, gestational diabetes, and diabetes of unknown type. *R*^2^ is coefficient of determination is a statistical measure in a regression model that determines the proportion of variance in the dependent variable that can be explained by the independent variable.

## Data Availability

The quantitative data used to support the findings of this study are available from the corresponding author upon reasonable request.

## Abbreviations

DKA: Diabetic ketoacidosis
DMSA: Type 1 Diabetes Mellitus Screening Acceptability
T1DM: Type 1 diabetes mellitus
GAD-7: Generalized Anxiety Disorder 7-item scale.

## Supplementary Materials

Supplementary 1 Informed consent script for participation and the Type 1 Diabetes Mellitus Screening Acceptability Assessment (DMSA) scale among general population.
