# Supplementary material for: The Psychometric Properties of the Type 1 Diabetes Mellitus Screening Acceptability Assessment (DMSA) Scale among General Population
Source: Pediatr Diabetes. 2024 Jul 25;2024:1286029. doi: 10.1155/2024/1286029 (PMC12017164; doi:10.1155/2024/1286029)
Supplement: Supplementary 1 — Informed consent script for participation and the Type 1 Diabetes Mellitus Screening Acceptability Assessment (DMSA) scale among general population. [file 1286029.f1.pdf]

Supplemental Material

**Informed Consent Script for Participation and the Type 1 Diabetes Mellitus Screening Acceptability Assessment (DMSA) Scale Among General Population (DMSA) Scale.**
**Instructions:**<br><br>The participant is required to read the script explaining Type 1 Diabetes Mellitus, its treatment, screening opportunities, and potential therapies to delay disease onset before answering the questions of the scale.<br><br>**Scoring:**<br><br>Each item is scored on a Likert scale ranging from 1 to 5, where a score of 5 indicated complete agreement and a score of 1 indicated complete disagreement. However, items Q9 and Q10 should be reverse scored. High score indicates higher acceptability.

Arabic Script:

ما هو مرض السكري من النوع الأول؟
مرض السكري من النوع الأول هو مرض غالبًا ما يبدأ في مرحلة الطفولة ويمكن أن يحدث في مرحلة الشباب. وهو ناتج عن تدمير الجهاز المناعي في الجسم للخلايا المنتجة للأنسولين في البنكرياس. وذلك بسبب عوامل جينية موروثة وعوامل أخرى، مثل الفيروسات. وهذا يعني أن أي طفل يمكن أن يصاب بمرض السكري من النوع الأول. الأطفال الذين يصابون بمرض السكري من النوع الأول يحتاجون إلى استخدام العلاج بحقن الانسولين للمحافظة على مستوى السكر في الدم مدى الحياة.
لقد قامت العديد من الدراسات البحثية حول العالم إلى اكتشاف الأفراد المعرضين لخطر الإصابة بمرض السكري من النوع الأول، أو قد تحمي البعض من الإصابة بالحموضة الكيتونية، أو تمكن العلماء على اكتشاف علاج يمنع أو يؤخر الإصابة بالسكري. ومع ذلك، فإننا نتفهم أن معرفة الأسرة بأن طفلها معرض لخطر الإصابة بـالسكري من النوع الأول قد يشكل عبئًا عاطفيًا. لذلك نحتاج إلى التأكد من أن هذا هو الشيء الصحيح الذي يجب القيام به في الوقت الحالي حيث أن العلاج الفعال حالياً هو الأنسولين فقط.

لماذا يعتبر الفحص المبكر مهم؟
يعتبر الفحص المبكر للسكري من النوع الأول من أحد الطرق الحديثة عالمياً لتتمكن من معرفة احتمالية إصابة الأشخاص في المستقبل بالسكري من النوع الأول. وتعتمد هذه الطريقة على أخذ عينة دم من الشخص وفحص ماذا كانت تحتوي على الأجسام المضادة للبنكرياس والتي تعتبر من أحد علامات بداية الهجوم المناعي المؤدي إلى الإصابة بالسكري. من خلال العثور على الأطفال المعرضين لخطر الإصابة بمرض السكري من النوع الأول في المستقبل، يمكننا مراقبة هؤلاء الأطفال عن قرب لمنع الإصابة بالحموضة الكيتونية. كما أن العثور على الأشخاص المعرضين للخطر يعني أن هؤلاء الأطفال يمكنهم اختيار الدخول في دراسات بحثية تختبر أدوية جديدة لتأخير بدء مرض السكري من النوع الأول.

ما هي وجهة نظرك حول الأسئلة التالية؟

1. **أفضل إجراء فحص الأطفال المبكر للسكري من النوع الأول لمعرفة احتمالية حدوثه**
   1. أتفق تمامًا
   2. أتفق
   3. محايد
   4. لا أتفق
   5. لا أتفق أبدا

2. **أعتقد أن القيام ببرنامج الفحص المبكر للسكري من النوع الأول للأطفال يعتبر مفيد ومنصف للعائلة**
   1. أتفق تمامًا
   2. أتفق
   3. محايد
   4. لا أتفق
   5. لا أتفق أبدا

أعتقد أن فحص الأطفال المبكر لسكري النوع الأول للأطفال يُمكن أن يُحَسِّن من فرص الرعاية أو التدخل المبكر

3.
1. أتفق تمامًا
2. أتفق
3. محايد
4. لا أتفق
5. لا أتفق أبدا

لدي استعداد للقيام بما يلزم لإجراء فحص الأطفال المبكر للسكري من النوع الأول (مثل الحضور للمواعيد، وأخذ عينات دم، وغيرها عند الحاجة)

4.
1. أتفق تمامًا
2. أتفق
3. محايد
4. لا أتفق
5. لا أتفق أبدا

أرغب بفحص طفلي المبكر للسكري من النوع الأول حتى لو كان على حسابي الخاص

5.
1. أتفق تمامًا
2. أتفق
3. محايد
4. لا أتفق
5. لا أتفق أبدا

إذا تم تنفيذ برنامج للفحص المبكر للسكري من النوع الأول للأطفال، أفضل الحصول على تفاصيل البرنامج من طبيب طفلي في سن مبكر

6.
1. أتفق تمامًا
2. أتفق
3. محايد
4. لا أتفق
5. لا أتفق أبدا

إذا تم تنفيذ برنامج للفحص المبكر للسكري من النوع الأول للأطفال، سأقوم بإجراء الفحص لأطفالي

7.
1. أتفق تمامًا
2. أتفق
3. محايد
4. لا أتفق
5. لا أتفق أبدا

إذا تم تنفيذ برنامج للفحص المبكر للسكري من النوع الأول للأطفال، سأقوم بمشاركة طفلي بنتائج الفحص بما يلائم فهمه

8.

1. أتفق تمامًا
2. أتفق
3. محايد
4. لا أتفق
5. لا أتفق أبدا

قد تؤثر إجراءات الفحص المبكر للسكري من النوع الأول لطفلي على حالتي النفسية بشكل سلبي

9.

1. أتفق تمامًا
2. أتفق
3. محايد
4. لا أتفق
5. لا أتفق أبدا

قد تؤدي النتيجة الايجابية للفحص المبكر للسكري من النوع الأول لطفلي إلى شعوري باختلاف نظرة المجتمع لعائلتي بشكل سلبي

10.

1. أتفق تمامًا
2. أتفق
3. محايد
4. لا أتفق
5. لا أتفق أبدا

English Script:
**What is Type 1 Diabetes?**<br>Type 1 diabetes is a condition often diagnosed in childhood or young adulthood. It results from the immune system attacking the insulin-producing cells in the pancreas, driven by a combination of genetic and environmental factors, including viruses. This autoimmune response makes the child susceptible to developing type 1 diabetes, necessitating lifelong insulin injection therapy for those affected.<br>Many research studies around the world have identified which individuals are at risk of developing type 1 diabetes, or prevent some from developing ketoacidosis, or enable scientists to discover a treatment that prevents or delays the development of diabetes.<br><br>**Why is screening for T1DM is important?**<br>Early screening for type 1 diabetes is a contemporary method that involves analyzing blood samples for antibodies linked to pancreas-related immune attacks, a precursor to diabetes. Identifying at-risk children allows for closer monitoring, reducing the risk of ketoacidosis. Moreover, these individuals may choose to participate in research studies testing new drugs to delay type 1 diabetes onset.

What is your opinion on the following questions?

1\. **I prefer screening my child for type 1 diabetes to find out how likely it is that they will get it.**

1. Strongly agree
2. Agree
3. No opinion
4. Disagree
5. Strongly disagree

2\. **I believe it is fair and helpful for the family to have a screening program for type 1 diabetes risk in children.**

1. Strongly agree
2. Agree
3. No opinion
4. Disagree
5. Strongly disagree

3. **I believe that early screening of children for type 1 diabetes risk can improve the chances for receiving early care or intervention**

1. Strongly agree
2. Agree
3. No opinion
4. Disagree
5. Strongly disagree

4. **I am willing to do whatever is needed for screening my child for type 1 diabetes (including attending appointments, taking blood samples, and more if needed).**

1. Strongly agree
2. Agree
3. No opinion
4. Disagree
5. Strongly disagree

5. **I would like to have my child screened for type 1 diabetes early, even if I have to pay for it myself**

1. Strongly agree
2. Agree
3. No opinion
4. Disagree
5. Strongly disagree

6. **If there is a program for early type 1 diabetes screening in children, I prefer to find details about it from my child's doctor at an early age.**

1. Strongly agree
2. Agree
3. No opinion
4. Disagree
5. Strongly disagree

7. **If there is program for early type 1 diabetes screening program, I will have my children checked.**

1. Strongly agree
2. Agree
3. No opinion
4. Disagree
5. Strongly disagree

If an early screening program for type 1 diabetes in children is carried out, I will share the screening results with my child as appropriate for their understanding.

8.

1. Strongly agree
2. Agree
3. No opinion
4. Disagree
5. Strongly disagree

The process of screening my child for type 1 diabetes risk may have a negative effect on my mental health.

9.

1. Strongly agree
2. Agree
3. No opinion
4. Disagree
5. Strongly disagree

If early screening for type 1 diabetes in my child comes back positive, I might feel negatively about how society thinks of my family.

10.

1. Strongly agree
2. Agree
3. No opinion
4. Disagree
5. Strongly disagree
